# Effects of Glycyrrhiza Glabra (Licorice) Root Extract on the Hormones, Serum Biochemicals, and Hematological Parameters in Dogs with Benign Prostatic Hyperplasia

**DOI:** 10.1155/2022/8962889

**Published:** 2022-12-09

**Authors:** Fatemeh Azadeh, Fatemeh Zahra Gharib, Seyed Mohammad Hosseini

**Affiliations:** ^1^Department of Veterinary Clinical Sciences, Babol Branch, Islamic Azad University, Babol, Iran; ^2^Department of Pathology, Babol Branch, Islamic Azad University, Babol, Iran

## Abstract

Benign prostatic hyperplasia (BPH) is the most common prostate disorder in non-neutered dogs and is often caused by androgenic stimulation or changes in the ratio of androgen to estrogen. Also, it is commonly seen in neutered older dogs. Clinical signs may not be presented, but persistent or intermittent hematuria, hemospermia, or a primary hemorrhagic discharge are usually reported. In this research, ethanolic extract of licorice root (EELR) was used as the treatment, which has various antioxidant and healing properties and can reduce lesions by establishing an antioxidant balance and reducing inflammation. For this experiment, 30 dogs of approximately the same age and weight were randomly divided into 6 groups of 5 each. The treatment groups received the EELR at doses of 10 and 20 mg/kg separately, while the control group received no medications. At the end of the 9-week treatment period, biochemical and hormonal factors were measured and analyzed by blood sampling. The results showed that the EELR has multiple positive effects on the serum biochemical indices and also positively affects hormone levels, while it can decrease the prostate-specific antigen (PSA) level in BPH dogs. In conclusion, the EELR can effectively reduce BPH-induced lesions without any side effects.

## 1. Introduction

The prostate is a gland behind the bladder that is located in the pelvis, directly below the rectum. Prostate disease is most common in humans, chimpanzees, and dogs, and rarely in cats. The disease is most common in 80% of neutered dogs over 5 years of age and 95% of neutered dogs over 9 years of age [1].

BPH is the most common prostate disease in non-neutered dogs [2]. As expected, no breed predisposition for BPH has been observed, and it may occur in almost any intact male dog [3]. One of the most important aspects in cases of BPH-related complications is to examine their correlation with urinary tract diseases, especially the diseases of the lower urinary tract, urinary tract infections, and kidney failure. Unlike prostatomegaly, which occurred in many cases, some of the dogs showed no clinical signs [4], so the protocol for BPH detection should be revised. Since surgery is not possible in many cases, medication therapy can be the choice of many patients to treat or alleviate the disease, among which is herbal medicine due to its natural antioxidants that can have a high medicinal potential [5]. This is an important issue because, generally, BPH damage can affect the healthy condition of other organs, such as the kidneys, and alter markers such as the creatinine and serum urea levels in the blood, both of which indicate the kidney's health [6].

Licorice has many active ingredients, which have shown some promising potentials, such as Glycyrrhizic acid, which has shown high anti-inflammatory, anticancer, and antioxidant potentials [7–9]. The leaf and root parts of this plant are rich in natural active ingredients, and the extracts of these parts of the plant are very rich in these active ingredients [10]. This plant also contains a high level of triterpenes, saponins, flavonoids, and several phenolic acids [11], which are proven to have antioxidant and anticancer effects [12, 13]. Glyceric acid and glycerin are useful for treating gastrointestinal ulcers [14]. The roots of the licorice plant also contain coumarin, flavonoids, essential oils, and plant sterols. In traditional medicine, this plant is used to treat muscle spasms and swelling, bronchitis, rheumatism, and arthritis [15].

One of the important points in cases of benign prostatic hyperplasia is the correlation of their primary disease with secondary urinary tract diseases, especially the lesions of the lower urinary tract. Urinary tract infections and kidney failure are only a small part of the pet owners' complaints when they visit a clinic. Since surgery is not possible in many cases, the medication for treating or alleviating the disease would be the first choice of many patients [5]. Regarding the association of BPH with renal problems, monitoring the renal health indices would be helpful [16, 17].

In this research, the effects of EELR on biochemical renal indices were investigated. Also, BPH following oxidative stress and hormonal misregulation were examined.

## 2. Materials and Methods

### 2.1. Study Design and Animal Grouping

BPH-suspected male dogs were physically examined, and a prostate test was performed to assess the parenchymal uniformity and the presence or absence of a cyst [18]. Then the susceptible dogs were identified due to factors such as nonuniformity, heterogeneous parenchyma, or the presence of cysts, which all greatly increase the risk of BPH. Finally, to ensure that the dogs were BPH-positive, an ultrasound was used, the prostate volume was examined, and BPH of the dogs was confirmed [19]. After finding 15 dogs with the BPH and 15 healthy dogs, which all had the same body condition (age and weight), they were randomly divided into 6 groups of 5 each. All dogs were kept under the same conditions. They all shared the same food and water. For the treatment, the ethanolic extract of licorice root (EELR) was administered orally to the dogs 3 days a week for 9 weeks. Finally, after 9 weeks, blood samples were taken from all the dogs from the cephalic vein. All dogs survived until the last day of the project. Male dogs were randomly divided into 6 groups of 5 each, including the control group (healthy dogs), the BPH group (sick dogs), the EELR group at a dose of 10 mg/kg (healthy dogs), the EELR group at a dose of 20 mg/kg (healthy dogs), the first treatment group of the EELR with the dose of 10 mg/kg (sick dogs), and the last treatment group of the EELR at a dose of 20 mg/kg (sick dogs) [20]. In the control and BPH groups, the dogs were fed water and food and did not receive any chemicals or medication. The ethical protocol of ARRIVE [21] was followed throughout the whole project, and the research ethics committees of the Islamic Azad University approved the project with the approval ID of IR.IAU.BABOL.REC.1400.083.

### 2.2. Extract Preparation

2 kg of freshly dried licorice root were purchased and powdered. Next, it was extracted by solving it in absolute ethanol, and then the extract was stored in a refrigerator at 4° Celsius until it was used [22].

### 2.3. Gas Chromatography Analysis of EELR

After extraction, 2 ml of the ethanolic extract of licorice root (EELR) was placed in a gas chromatography machine (Shimadzu GCMS-TQ8040 NX) and analyzed. The reported substances were specified by comparing their spectra with those in Wiley and the NIST/EPA/NIH34-44 spectral mass libraries [23, 24].

### 2.4. Blood and Serum Tests

All the blood and serum markers were assessed by the autoanalyzer. The hormones and stress markers were also estimated with special kits.

### 2.5. Statistical Analysis

Blood factors were analyzed using SPSS software version 26 and a one-way ANOVA with a Tukey post hoc test. The minimum significance level was set to *P* < 0.05 [22].

## 3. Results

### 3.1. GC-MS of EELR

The ethanolic extract of licorice root was placed inside a gas chromatographic machine, and the results were read after 3 repetitions. Components that were less than 1% were removed from the table and merged as Others (Table 1).

### 3.2. Complete Blood Count (CBC)

The number of red blood cells (RBC) in the BPH group was higher than that of the control group, while in the BPH + EELR groups, a slight decrease was observed compared to the BPH group. The hemoglobin (HGB) was not significantly different between the groups, but it should be noted that its number went down in the EELR groups compared to the control. The hematocrit (HCT) in the BPH group was higher than that of the control group. The levels of other factors were also elevated in the BPH group compared to the control group, but no significant difference was seen (Table 2).

Platelet levels were increased in the BPH groups compared to the control group, which then decreased in the BPH + EELR10 group. Also, in the EELR groups, the EELR20 showed a moderate increase in platelet levels compared to the control group. PCT levels were not significantly different in all groups. The level of MPV in the BPH group showed an increase compared to the control group, while in all groups that were affected by the EELR, we saw a clear fall compared to the control and BPH groups. PDW levels did not differ significantly between the groups (Table 3).

### 3.3. White Blood Cell Count (WBC)

The WBC count in the BPH group was lower compared to the control group. However, in all groups that were affected by the EELR, we saw a moderate increase in the WBC compared to both the control and BPH groups. There was no significant difference in the neutrophil percentages between all groups. On the other hand, slight increases in the percentages of lymphocytes, monocytes, and eosinophils were observed in the BPH group compared to the control group, which were also not significant (Table 4).

### 3.4. Serum Biochemical Parameters

The levels of both albumin and total protein (TP) indices were slightly increased in the BPH group compared to the control group, but no significant change was observed. Regarding insulin, we saw a decrease in its level in the BPH group compared to the control group, which was even lower in the EELR-receiving groups, which again was not significant. Consequently, glucose levels did not show a significant difference between the groups, but a slight increase in the glucose level of the BPH + EELR10 group was observed. ALT and AST levels were increased in the BPH group compared to the control group, while they were decreased in the BPH + EELR groups compared to the BPH group moderately (Table 5).

### 3.5. Kidney Parameters

The blood urea nitrogen (BUN) level in the BPH group was increased compared to the control group, but in the BPH + EELR20 group, it was substantially reduced. There was no significant difference in the uric acid levels between the groups. The creatinine level was also increased in the BPH group compared to the control group, while it was decreased in both treatment groups compared to the BPH group. The amounts of calcium and phosphorus in the groups were not significantly different from each other (Table 6).

### 3.6. General Cell Damage Indices

Levels of both LDH and CPK indices were increased in the BPH group compared to the control group. Meanwhile, a decrease in both of these markers was seen in the treatment groups, which was more in the BPH + EELR20 group (Table 7).

### 3.7. Hormones and PSA

**Figure 1:**
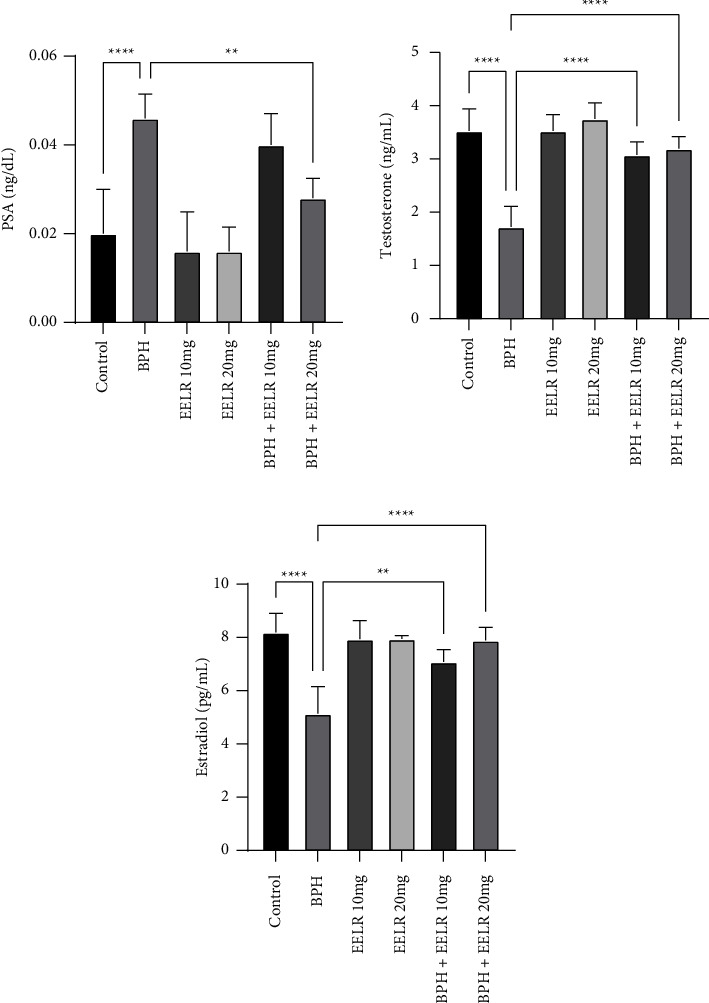
Comparison between the hormone indices. ^*∗∗*^*P* < 0.01, and ^*∗∗∗∗*^*P* < 0.0001: significant compared to the BPH group. All results are expressed as the mean ± standard error. *N* = 5.

The PSA level in the BPH control group was significantly higher than the control group (*P* < 0.0001). In the BPH + EELR20 group, a significant fall in the PSA level was observed compared to the BPH group (*P* < 0.01). However, there was not a significant difference between the EELR10 and the BPH group. Also, no significant difference was observed between the EELR-only and control groups. The estradiol and testosterone levels showed a significant decrease (*P* < 0.0001) in the BPH group compared to the control group. In both cases, the EELR was able to increase the levels of these hormones, with the EELR20 increasing both significantly (*P* < 0.0001) and bringing them closer to those of the control group (Figure 1).

### 3.8. Oxidative Stress Indices

**Figure 2:**
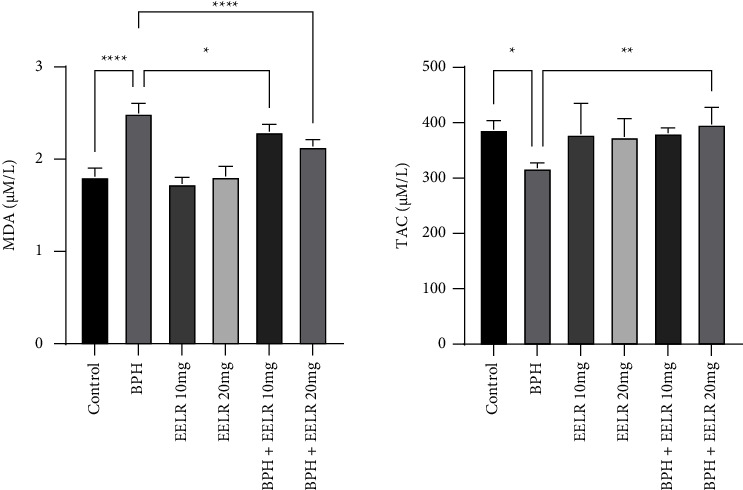
Comparison between the tissue stress marker indices. ^*∗*^*P* < 0.05, ^*∗∗*^*P* < 0.01, and ^*∗∗∗∗*^*P* < 0.0001: significant compared to the BPH group. All results are expressed as the mean ± standard error. *N* = 5.

After examining the levels of serum stress markers, it was observed that the level of malonaldehyde (MDA) in the dogs with BPH was significantly higher than that of the control group. In the EELR10 treatment group, it was seen that the MDA level was significantly decreased (*P* < 0.05) compared to the BPH group, and in the EELR20 treatment group, this increase was even more (*P* < 0.0001). The total antioxidant capacity (TAC) level in the BPH group was significantly (*P* < 0.05) lower than the control group, but significantly higher in the BPH + EELR20 group compared to the BPH group (*P* < 0.01) (Figure 2).

## 4. Discussion

In this study, the effect of ethanolic extract of licorice root (EELR) at the two doses of 10 mg/kg and 20 mg/kg on dogs with BPH was investigated, and in addition, the toxicity of these two doses was examined separately. The licorice extract had been shown to be significantly effective in the prostate cancer cell line [25]. The effects of the different natural substances were previously studied on BPH human patients [26, 27], and it was shown that they could be effective. Even a positive anticancer effect was seen in an earlier study of the licorice extract [28].

Initially, after the GC-MS result of the EELR, it was observed that this extract has many flavonoids and antioxidants, each of which has the potential to decrease inflammation and tissue damage such as hyperplasia [29]. It was shown that the upper part of this plant has many beneficial substances, and now it is shown that the root part of it has the potential, too [30]. There were some critical compounds like caffeine [31], palmitic acid [32], oleic acid [33], and linoleic acid [34] in the EELR, most of which have proven regenerative potentials. As an example, oleic acid was shown to have anticancer and antiproliferative characteristics [23]. Moreover, palmitic acid was shown to have antiproliferative potentials in an earlier study [35]. Also, after 9 weeks, no adverse effects from the administration of the EELR were seen in any dog.

Regarding the hematological parameters, it was observed that the EELR in both doses didn't cause a significant change between the dogs with BPH and the healthy dogs, as it had in the previous studies [1, 36]. This issue demonstrated that there was no blood toxicity following the consumption of the EELR. In the matter of the serum indices, after the platelet level in the BPH group was increased compared to the control group, a decrease in the EELR group was observed that could be the result of the natural regenerative compounds of the EELR (Table 1). Also, the PCT, MPV, and PDW levels showed no significant differences in the groups. The same trend happened to the WBCs, and their percentages didn't change significantly (Table 4), which was similarly in line with the previous studies [1, 36].

As was seen in an earlier study [1], the level of albumin, as the main protein of plasma and one of the general health indices of the body, wasn't significantly correlated to the BPH, and despite a slight decrease in the BPH group compared to the control group, it didn't change significantly in this study (Table 5). The number of liver enzymes in the BPH group was increased compared to the control group, which indicated that BPH can affect those indices. However, the changes were not significant; thus, they did not have a huge impact. Even that slight elevation of the liver indices could be caused by the natural cell-damaging effects of BPH [36]. In addition, the anti-inflammatory potential of insulin was observed [37]. However, despite the nature of BPH, which is a kind of asymptomatic inflammatory prostatitis [38], no sign of a significant alteration of insulin was observed in this research. A possible reason for that could be related to the substances in EELR, which include a lot of sugars and eventually downregulate the insulin to keep the serum glucose level steady. Fortunately, despite the moderate changes in insulin levels, no significant change was observed in glucose levels across all groups.

As the previous study said [1], the effect of BPH on renal parameters was undeniable, as was observed in this study. However, the changes were not significant between the control, the BPH, and the EELR groups. It was expected that the BUN level would increase significantly in the BPH group compared to the control group [36], but nothing was seen (Table 6). Also, the other possible reason for that issue could be the dogs' lack of a nutritious diet. After all, it is obvious that the BPH can elevate the kidney indices, just not enough to be concerned about them. It could hardly be seen that the creatinine and uric acid levels were increased in the BPH group compared to the control group, which meant that some renal damage occurred, but again, not too much to be concerned about [39]. It is also worth mentioning that the amounts of calcium and phosphorus were not changed significantly.

The levels of both LDH and CPK markers in the BPH group were increased compared to the control group, which indicated that the BPH could cause cell destruction and muscle damage [40]. However, consumption of the EELR caused an increase in both of these markers, which could be the result of cell regeneration or reduced cell destruction.

The most important indicator for detecting and staging BPH is the PSA level, which was significantly higher in the BPH group compared to the control dogs (*P* < 0.0001) as was shown in the previous study [25, 41]. In the EELR20 treatment group, we saw a significant decrease (*P* < 0.01) in the PSA level compared to the BPH group. It seems that the EELR, with its antioxidant [42] and regenerative [43] properties, was able to reduce the amount of PSA in sick dogs by affecting the prostate tissue [44].

The levels of both estradiol and testosterone were significantly decreased in the BPH group compared to the control group. Both of these hormones play a key role in BPH-patients [45]. The estradiol was decreased because of the probable rise of the inhibitory pathways. As it was seen, the testosterone level was fallen due to the BPH, which caused the stimulating cells to proliferate and stop functioning correctly, and at the same time trigger the signaling pathways that led to the inhibition of the estradiol [46, 47]. Reduction of the testosterone level can also cause spermatogenesis dysfunction, which eventually leads to a worse clinical prognosis [48]. It should be noted that both of these hormones are important sex hormones, which were significantly elevated in the EELR20 group (*P* < 0.0001) due to the regenerative potentials and antioxidant properties of the EELR.

Finally, the serum MDA level was examined to estimate the amount of oxidative abnormality, which was significantly higher in the BPH group compared to the control group due to the cell destruction and tissue stress caused by reducing the ROS, which was consistent with the previous study [49]. However, that increase was downregulated by the EELR20 administration significantly due to the natural antioxidants in the EELR. In addition, the TAC level had a negative correlation with the MDA level, and it was significantly lower in the BPH group compared to the control group (*P* < 0.05) [50, 51]. Similarly, a significant rise in the TAC level was observed in the EELR20-treated dogs, which demonstrated a shining antioxidant potential. Based on earlier research, it was observed that tadalafil, as a strong antiproliferative agent, was not able to regulate the inflammatory mediators in the BPH dogs [52], while the EELR, as a natural agent, managed to positively reduce ROS levels. It has been shown before that natural substances can effectively reduce oxidative stress and inflammation [22].

## 5. Conclusion

Overall, it can be stated that the EELR can reduce the rate of prostate lesions in BPH effectively because of its anti-inflammatory and antioxidant properties, which are boosted by its natural flavonoid compounds, and also because it a significant level of omega-6 fatty acids. Regulating renal function in the excretion of substances and serum markers was another result of the administration of EELR. Finally, no toxicity or adverse effect was observed in the dogs at either dose.

## Figures and Tables

**Table 1:** The percentages of active ingredients in the EELR that were analyzed by the GC-MS machine.

Chemical constituents	Retention time	Peak area (%)	Molecular weight	Molecular formula
Caffeine	17.486	1.23	194.19	C_8_H_10_N_4_O_2_
Palmitic acid	17.943	7.68	256.42	C_16_H_32_O_2_
(Z,Z)-9,12-Octadecadienoic acid	19.560	4.43	280.45	C_18_H_32_O_2_
Oleic acid	19.612	3.60	282.46	C_18_H_34_O_2_
Linoleic acid	20.160	12.83	280.45	C_18_H_32_O_2_
Ethyl oleate	20.212	11.77	310.51	C_20_H_38_O_2_
Cyclotetradecane	21.240	2.03	196.37	C_14_H_28_
Hemipic acid	23.360	2.29	226.18	C_10_H_10_O_6_
7-Pentadecyne	24.469	13.01	208.38	C_15_H_28_
1,1,3,3,5,5,7,7,9,9,11,11,13,13,15,15-Hexadecamethyloctasiloxane	24.665	27.07	577.2	C_16_H_48_O_7_Si_8_
Others	—	14.06	—	—

**Table 2:** Comparison between the RBC parameters in each group. All results are expressed as the mean ± standard error. There was no significant difference between the groups. *N* = 5.

	RBC (×10^6^/*μ*L)	HGB (g/dL)	HCT (%)	MCV (fL)	MCH (pg)	MCHC (g/dL)	RDW (%)
Control	6.33 ± 0.35	13.30 ± 0.37	37.50 ± 0.46	61.92 ± 1.13	21.08 ± 0.26	34.04 ± 0.28	12.08 ± 0.24
EELR10	6.29 ± 0.48	13.08 ± 0.37	37.84 ± 1.47	61.94 ± 2.41	21.08 ± 0.14	34.10 ± 0.77	12.06 ± 0.2
EELR20	6.32 ± 0.36	12.93 ± 0.46	37.75 ± 1.11	61.70 ± 1.49	21.40 ± 0.17	34.05 ± 0.39	12.13 ± 0.35
BPH	6.48 ± 0.19	13.40 ± 0.38	38.82 ± 1.1	63.76 ± 1.16	21.62 ± 0.24	34.34 ± 0.25	12.18 ± 0.29
BPH + EELR10	6.47 ± 0.19	13.50 ± 0.27	38.58 ± 2.10	62.20 ± 2.01	21.75 ± 0.6	34.33 ± 0.45	12.20 ± 0.40
BPH + EELR20	6.14 ± 0.38	12.92 ± 0.49	37.66 ± 0.96	62.62 ± 1.59	21.08 ± 0.24	33.78 ± 0.60	12.08 ± 0.26

RBC, red blood cell; HGB, hemoglobin; HCT, hematocrit; MCV, mean corpuscular volume; MCH, mean corpuscular hemoglobin; MCHC, mean corpuscular hemoglobin concentration; RDW, red cell distribution width.

**Table 3:** Comparison between the platelet parameters in each group. All results are expressed as the mean ± standard error. There was no significant difference between the groups. *N* = 5.

	Platelets (×10^3^/*μ*L)	PCT (%)	MPV	PDW
Control	355.00 ± 52.72	0.22 ± 0.04	7.64 ± 0.4	16.86 ± 0.32
EELR10	368.00 ± 51.54	0.26 ± 0.03	7.04 ± 0.27	16.14 ± 0.65
EELR20	386.25 ± 45.49	0.25 ± 0.03	7.10 ± 0.33	16.78 ± 0.71
BPH	399.00 ± 26.33	0.25 ± 0.03	7.94 ± 0.24	16.28 ± 0.37
BPH + EELR10	370.50 ± 60.49	0.24 ± 0.02	7.45 ± 0.15	16.35 ± 0.49
BPH + EELR20	392.80 ± 49.00	0.25 ± 0.02	7.12 ± 0.26	16.06 ± 0.38

PCT, plateletcrit; MPV, mean platelet volume; PDW, platelet cell distribution width.

**Table 4:** Comparison between the WBC parameters in each group. All results are expressed as the mean ± standard error. There was no significant difference between the groups. *N* = 5.

	WBC (×10^3^/*μ*L)	Neutrophil (%)	Lymphocyte (%)	Monocyte (%)	Eosinophil (%)
Control	8.82 ± 0.49	63.60 ± 5.24	22.00 ± 3.00	4.40 ± 0.81	9.80 ± 4.01
EELR10	9.22 ± 0.75	64.20 ± 4.90	24.80 ± 2.99	4.80 ± 0.86	5.60 ± 1.08
EELR20	9.33 ± 0.74	68.75 ± 3.28	20.25 ± 3.25	4.00 ± 0.71	6.00 ± 2.16
BPH	9.68 ± 0.58	71.40 ± 3.93	19.40 ± 1.91	5.60 ± 0.51	9.80 ± 1.93
BPH + EELR10	9.28 ± 1.03	68.50 ± 8.65	26.75 ± 4.09	6.00 ± 0.91	3.50 ± 1.66
BPH + EELR20	9.24 ± 0.69	64.00 ± 4.2	25.60 ± 2.62	4.80 ± 0.49	6.20 ± 1.07

**Table 5:** Comparison between the biochemical parameters in each group. All results are expressed as the mean ± standard error. There was no significant difference between the groups. *N* = 5.

	Albumin (g/dL)	Total protein (g/dL)	Insulin (mU/L)	Glucose (mg/dL)	ALT (IU/L)	AST (IU/L)
Control	3.26 ± 0.07	7.00 ± 0.36	15.18 ± 1.65	85.40 ± 7.08	49.20 ± 5.13	38.20 ± 6.27
EELR10	3.16 ± 0.02	7.20 ± 0.30	12.14 ± 2.39	81.40 ± 2.20	47.40 ± 5.83	32.20 ± 3.25
EELR20	3.20 ± 0.04	7.08 ± 0.26	11.28 ± 1.60	80.75 ± 3.71	51.00 ± 3.49	38.75 ± 5.94
BPH	3.30 ± 0.04	7.66 ± 0.28	13.12 ± 1.02	85.20 ± 5.99	58.00 ± 9.56	42.00 ± 6.91
BPH + EELR10	3.38 ± 0.06	6.83 ± 0.38	10.63 ± 2.24	94.00 ± 4.74	46.25 ± 4.42	39.00 ± 4.53
BPH + EELR20	3.18 ± 0.17	7.38 ± 0.11	10.60 ± 1.03	80.40 ± 3.96	46.60 ± 7.26	40.80 ± 3.38

**Table 6:** Comparison between the kidney indices in each group. All results are expressed as the mean ± standard error. There was no significant difference between the groups. *N* = 5.

	BUN (mg/dL)	Uric acid (mg/dL)	Creatinine (mg/dL)	Calcium (mg/dL)	Phosphorus (mg/dL)
Control	20.64 ± 2.68	0.16 ± 0.02	1.07 ± 0.04	10.10 ± 0.49	4.48 ± 0.18
EELR10	23.00 ± 2.61	0.14 ± 0.02	1.1 ± 0.03	9.48 ± 0.28	4.74 ± 0.24
EELR20	19.38 ± 3.16	0.13 ± 0.03	1.09 ± 0.03	9.45 ± 0.21	4.28 ± 0.3
BPH	26.06 ± 1.75	0.14 ± 0.02	1.27 ± 0.06	9.78 ± 0.35	4.42 ± 0.2
BPH + EELR10	26.33 ± 3.51	0.18 ± 0.05	1.11 ± 0.09	9.83 ± 0.39	4.45 ± 0.56
BPH + EELR20	21.86 ± 1.04	0.18 ± 0.02	1.16 ± 0.09	9.92 ± 0.3	4.46 ± 0.17

**Table 7:** Comparison between the inflammation and cell damage indices in the serum of each group. All results are expressed as the mean ± standard error. There was no significant difference between the groups. *N* = 5.

	LDH (U/L)	CPK (U/L)
Control	109.20 ± 13.94	146.20 ± 29.81
EELR10	123.60 ± 24.16	125.40 ± 16.46
EELR20	133.00 ± 12.19	133.50 ± 15.14
BPH	117.40 ± 18.89	172.60 ± 28.83
BPH + EELR10	114.25 ± 14.36	128.50 ± 29.71
BPH + EELR20	113.60 ± 23.15	106.00 ± 15.59

## Data Availability

The data are available on a reasonable request from the corresponding author.
